# Fabrication of Disposable Thin-Film Electrodes via Shadow Mask Technique for Sensitive Point-of-Care Detection of C‑Reactive Protein, a Sepsis Biomarker

**DOI:** 10.1021/acsomega.5c01453

**Published:** 2025-10-27

**Authors:** Mahsa Kalantar, Ali Hossein Rezayan, Hassan Hajghassem, Reza Askari Moghadam, Hedayatollah Ghourchian, Amir Mohamadsharifi

**Affiliations:** 1 School of Life Science Engineering, College of Interdisciplinary Science and Technology, 48425 University of Tehran, Tehran 1439957131, Iran; 2 School of Intelligent Systems, College of Interdisciplinary Science and Technology, 48425 University of Tehran, Tehran 1439957131, Iran; 3 Sorbonne Université, CNRS, INSERM, Laboratoire d’Imagerie Biomédicale, LIB, F-75006 Paris, France; 4 Laboratory of Bioanalysis, Institute of Biochemistry & Biophysics, University of Tehran, 13145-1384 Tehran, Iran

## Abstract

Point-of-care testing for sepsis refers to diagnostic technologies that rapidly and effectively detect sepsis using specific biomarkers. The development of thin-film electrodes (TFEs) for biosensing applications has been frequently reported in recent years. Notably, TFEs have been employed to develop biosensors for C-reactive protein (CRP), a key biomarker of sepsis. This study presents a straightforward and rapid method for fabricating TFEs on substrates using a shadow mask technique. The process employs commercially available polyvinyl chloride sticker film, serving as a disposable shadow mask, to create a three-electrode system (working electrode (WE), counter electrode (CE), and reference electrode (RE) on a poly­(methyl methacrylate) substrate. The sequential deposition of 20 nm of titanium (Ti) and 120 nm of gold (Au) formed the WE and CE, while the RE was fabricated by depositing titanium and silver (Ag). The fabricated TFEs were characterized by field emission scanning electron microscopy, atomic force microscopy, and energy-dispersive X-ray spectroscopy analyses, revealing notable differences in surface morphology and elemental composition. A specific aptamer for the CRP biomarker is immobilized on the surface of a TFE. The electrochemical biosensor demonstrated a linear range of 0.02–0.9 ng/mL and a limit of detection of 6.5 pg/mL, indicating high sensitivity for detecting the CRP biomarker.

## Graphical abstract

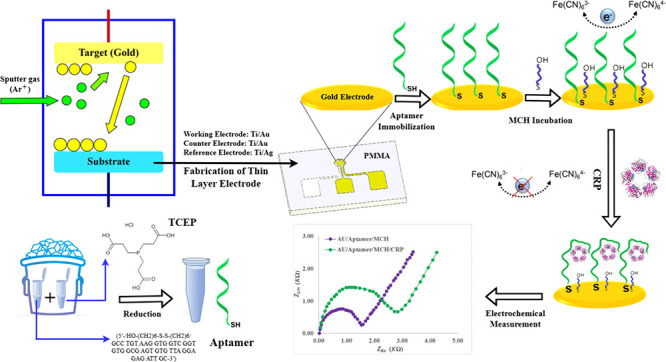

## 1 Introduction

Over the past few decades, microelectrodes have been widely employed in integrated sensing platforms, often referred to as micrototal analytical systems (μTAS), including interdigitated electrodes (IDEs),^1^ electrochemical sensors,^2^ field-effect transistors (FETs),^3^ and related devices. Most of these electrodes are fabricated using screen printing,^4^ photolithography,^5^ or shadow mask lithography (SML).^6^ The choice of fabrication method depends on multiple factors, as each technique has distinct advantages and limitations. Traditionally, bulk electrodes represent the earliest class of electrodes employed in electrochemical processes.^7^ They are accurate and reusable but require a troublesome cleaning process after each use that require consumption of biohazard chemical.^8^ Hence, they are not applicable to be employed in point-of-care diagnosis.^9^ The second type of electrodes mainly used for electrochemical sensors are screen-printed electrodes (SPE), which are commercially available. SPEs are widely used in various electrochemical sensors. These electrodes are fabricated by applying a semiliquid paste, which is a composite containing conductive micro/nanoparticles dispersed in a polymer matrix, onto a substrate.^10,11^ The substantial thickness of films produced by screen printing can hinder the integration of complex microfluidic structures via lamination or bonding. Additionally, the composite nature of SPEs comprising gold particles, binders, and impurities reduces intrinsic conductivity, thereby compromising sensitivity relative to pure bulk or thin-film electrodes. While SPEs exhibit lower intrinsic conductivity, their greater thickness often yields lower sheet resistance than thin-film electrodes (TFEs).^12^ Further drawbacks of SPEs include limited durability, environmental susceptibility, reduced current capacity, and surface roughness.^13^ Surface modification techniques, such as incorporating graphene, carbon nanotubes, or metal nanoparticles via chemical or physical deposition, have been employed to enhance conductivity, improve detection limits, and minimize redox interferences.^14,15^

In contrast, TFEs have only recently become commercially available for biosensor applications and offer several advantages over SPEs. Typically fabricated from high-purity materials, they are most often produced via photolithography and physical vapor deposition (PVD) techniques, such as sputtering.^16^ It is possible to integrate TFEs with microfluidic devices,^17^ without any risk of leakage, owing to their minimal height, which typically does not exceed 1 μm.^18^ TFEs are fabricated on glass,^19^ silicon,^20^ Pyrex,^21^ rigid polymers such as cycle-olefin polymer,^22^ and polycarbonate (PC), and flexible polymers such as polyethylene naphthalate (PEN),^23^ and polyaminoanthraquinone (PAAQ).^24^ Moreover, the deposited material used in PVD is versatile and can be chosen easily by changing the sputtering target between gold,^25^ platinum,^26^ nickel oxide,^27^ and indium tin oxide.^28^ Although TFEs exhibit higher sheet resistance due to their reduced thickness, they retain superior intrinsic conductivity because the gold forms a continuous, high-purity metallic layer. Furthermore, their notable sensitivity and lower detection limits make them particularly applicable in lab-on-chip (LOC) devices.^21,29^ A reported drawback of TFEs is the complexity and expense of their fabrication process. Unlike SPEs, which can be batch-produced, TFEs often require significant expertise and specialized facilities, including clean rooms for photolithography steps.^30,31^

One of the key challenges in utilizing TFEs lies in their dependence on substrate material. Conventional fabrication techniques, such as photolithography, require precise control over the substrate’s physical and chemical properties, which can limit the flexibility and compatibility of TFEs across diverse applications.^32^ Additionally, the intrinsic characteristics of the substrate can directly influence the electrical and mechanical performance of TFEs.^33^ Moreover, fabricating three-electrode cells from different materials presents notable challenges, often requiring multiple deposition steps and repeated lithography processes. Traditional photolithography, for example, encounters difficulties when applied to substrates such as paper, as the wet processing solutions can saturate the material and disrupt pattern formation.^34^ Similarly, certain photolithographic procedures necessitate the use of various solvents, resulting in time-consuming steps and potential substrate damage.^35,36^ These limitations highlight the need for alternative fabrication strategies for TFEs that are simpler, faster, and accessible to nonspecialists.

egardless of the electrode type, These can be applied in the diagnosis and prognosis of diseases through the quantitative detection of various biomarkers, including those associated with sepsis.^37^ Sepsis is a life-threatening condition that arises when the immune system mounts a rapid and intense response to infection, releasing proteins and other mediators to combat the invading pathogens.^38^ If this response becomes dysregulated, it can lead to widespread systemic inflammation. Although bacterial infections remain the primary cause of sepsis,^39^ viral pathogens such as COVID-19,^40^ and influenza,^41^ as well as fungal infections^42^ are also recognized triggers.^43^ Common symptoms of sepsis include fever, tachycardia, and shortness of breath, all of which require urgent medical intervention.^44^ The concept of point-of-care (POC) testing refers to performing medical diagnostics and analyses at or near the patient’s location, eliminating the need to send samples to centralized laboratories.^45^ Consequently, the rapid and accurate detection of sepsis biomarkers at the early stages is critical for effective patient management in POC-based diagnostic devices.^46^

Many current research efforts focus on developing biosensors that are simple to fabricate while offering sufficient chemical and physical stability for point-of-care applications, a feature considered a major advantage.^47^ Clinically important biomarkers for sepsis include procalcitonin (PCT),^48^ C-reactive protein (CRP),^49,50^ tumor necrosis factor-alpha (TNF-α),^51^ Interleukin-6 (IL-6),^52^ and pancreatic stone protein (PSP).^53^ Among these, CRP is recognized as a particularly important biomarker for the diagnosis of sepsis.^54^

This research focuses on point-of-care diagnosis with an emphasis on sepsis biomarkers. This study proposed a simplified approach to making multimaterial, thin-film, disposable electrodes using a shadow mask technique applied to a PMMA substrate. This innovative method avoided the expensive and time-consuming photolithography steps, instead employing available PVC sticker film as a disposable shadow mask and the result was a three-electrode system. Also, it compared its performance with commercial products commonly utilized in biosensors. We discussed the advantages and limitations of the newly developed electrode. To validate its functionality, the electrode was employed in an electrochemical sensing experiment targeting the CRP biomarker as a critical biomarker of sepsis.

## 2 Materials and Methods

### 2.1 Material and Apparatus

PMMA (Year Long Industrial Co., Taiwan), PVC (Sigma-Aldrich Co., Germany), Titanium sputtering target, diameter 2.00 in. Thickness 0.25in (Sigma-Aldrich Co., Germany), Gold High-purity (99.999%) (Sigma-Aldrich Co., Germany), and Silver Sputtering Target (99.99%) (Heegermaterials, USA) were purchased. Disposable screen-printed electrodes were acquired from Metrohm DropSens Company.

The aptamer was synthesized by Pishgam Biotech Co., Ltd. (Tehran, Iran). 6-mercapto-1-hexanol (MCH), potassium ferricyanide (K_3_ [Fe (CN) _6_]), ferrocyanide (K_4_ [Fe (CN) _6_]), and phosphate-buffered saline (PBS) were procured from Sigma-Aldrich (Munich, Germany). CRP protein (MBS390133) was obtained from MyBiosource Co. Tris (2-carboxyethyl) phosphine hydrochloride (TCEP) was purchased from Molekula Co.

Electrochemical measurements, including cyclic voltammetry (CV) and electrochemical impedance spectroscopy (EIS), were performed using a potentiostat/galvanostat (IVIUMSTAT, Ivium Technologies). A CO_2_ laser cutter (Glowforge Pro, 45 W) and a cutter plotter (AOL-1612PAS; William International CNC Technology) were used for fabrication. Thin films were deposited using a DC magnetron sputtering system (model MSS160, ACECR Co.). Film thickness was measured with a stylus profilometer (Dektak 150, Bruker Co). Surface roughness was characterized by atomic force microscopy (AFM; ENTEGRA, NT-MDT). Field-emission scanning electron microscopy (FESEM; TESCAN MIRA3) was performed, and elemental composition was determined by energy-dispersive X-ray spectroscopy (EDS).

### 2.2 Methods

#### 2.2.1 Fabrication of Thin Film Electrode Cell

In this study, PMMA served as a rigid substrate,^55^ for the electrodes. A sheet of PMMA,^56^ was precisely cut into small 3 × 2 cm pieces using a CO_2_ laser cutter.^57^ The electrode designs were crafted in CorelDraw software.^58^ The system’s design incorporates a three-electrode configuration consisting of a counter electrode (CE), a working electrode (WE), and a reference electrode (RE). In the designed structure, the WE features a 2 mm diameter and is encircled by a ring arc with a width of 300 μm, set within a 400 μm space. Positioned opposite the CE is another arc of a ring, shorter in length, which functions as the RE. The electrodes are centrally positioned within the rectangular layout to ensure symmetrical geometry. Each electrode is connected by a 0.5 mm-wide conductive track extending to the edge of the substrate, ensuring straightforward electrical access. The material differences between the working/counter electrodes (WE/CE) and the reference electrode (RE) required dividing the overall layout into two distinct sections. The first layer comprises the WE and CE, while the second layer encapsulates these components and incorporates the RE geometry.^59^

A commercially available PVC sticker film was employed as a disposable shadow mask for all deposition stages. The electrode geometries were precisely patterned and cut into the PVC film using a cutter plotter, with parameters optimized to produce smooth, residue-free edges.^60^ The first mask was applied to a PMMA substrate, exposing the regions designated for the working electrode (WE) and counter electrode (CE). Thin films of titanium (20 nm) and gold (120 nm) were then sequentially deposited onto the CE and WE via sputtering. In contrast, the reference electrode (RE) was fabricated by sputter-depositing titanium and silver layers.^61^

Figure 1 presents a detailed step-by-step schematic of the electrode fabrication process, utilizing precut PVC stickers patterned with the desired electrode geometries. These stickers adhere to the substrate surface, acting as shadow masks, and are positioned on a PMMA base for subsequent deposition steps. The initial mask precisely outlines the geometry of the working electrode (WE), counter electrode (CE), and reference electrode (RE). The mask is applied to the prepared PMMA substrate, with the regions corresponding to the working electrode (WE) and counter electrode (CE) carefully removed, while leaving the reference electrode (RE) section intact. Subsequently, layers of titanium (20 nm) and gold (120 nm) are deposited: the titanium layer enhances adhesion to the substrate, while the gold layer forms the actual electrode. The thickness of these layers is continuously monitored using an embedded crystal sensor during the deposition process. Following this, the RE area in the first mask was exposed, and a second mask designed exclusively for the RE was precisely aligned and adhered over the first layer. This configuration ensured that the previously fabricated WE and CE were completely covered while leaving an opening for silver deposition onto the RE. The adhesive property of the PVC mask prevented displacement of the underlying layer, thereby facilitating accurate alignment. A 20 nm titanium adhesion layer was first sputtered, followed by a 120 nm silver layer, completing the fabrication of the RE.

**Figure 1:**
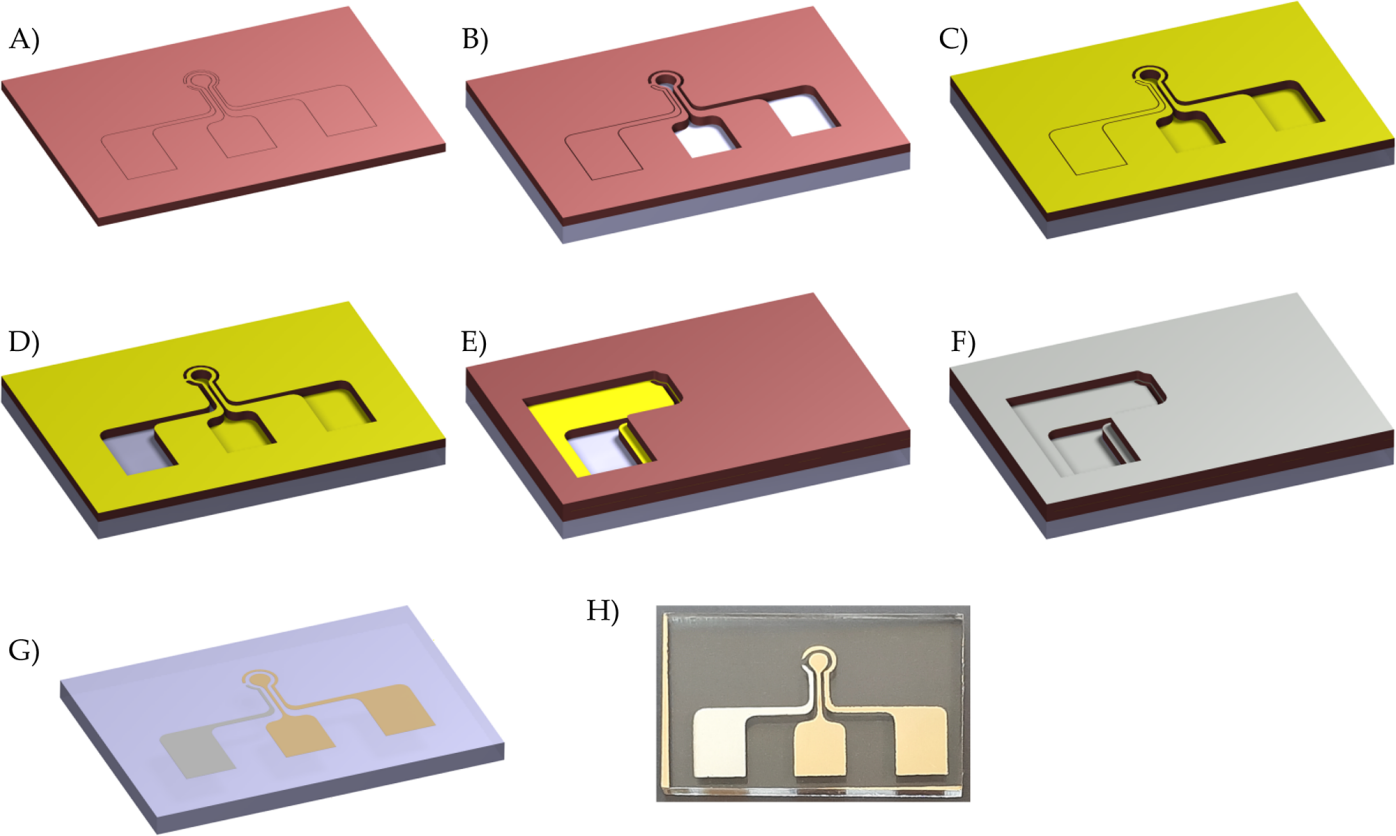
Geometrical specifications of the triplet electrode cell along with the sequential fabrication steps: (A) cutting the first PVC sticker mask, (B) removing the WE and CE windows and laminating the first mask onto the substrate, (C) sputter-depositing titanium and gold onto the exposed areas, (D) opening the RE window, (E) aligning and adhering the second mask over the first mask with an open space for the RE, (F) sputter-depositing titanium and silver onto the RE, (G) removing both masks, and (H) actual image of the fabricated electrode set.

#### 2.2.2 Fabrication of the Micro Reservoir

A 5 mm-thick PMMA sheet was used to fabricate the microreservoir. A CO_2_ laser precisely cut a rectangular structure with external dimensions of 30 × 11 mm, incorporating a central chamber measuring 13 × 6 mm to define the contact area between the sample solution and the electrode surfaces. A double-sided adhesive layer, 70 μm thick and matching the same geometry, was subsequently fabricated using the CO_2_ laser and applied to secure the reservoir to the electrodes, thereby creating a leak-proof cavity. This configuration provides a controlled, low-volume liquid environment over the electrodes.

#### 2.2.3 Electrochemical Performance

Cyclic voltammetry (CV) and electrochemical impedance spectroscopy (EIS) were employed to characterize the fabricated sensors. The electrolyte consisted of 2 mM potassium ferro-/ferricyanide.^62^ All experiments were conducted in triplicate, and the mean values of these repetitions were reported as the final results. To avoid potential inaccuracies, data from the first CV cycle were excluded from analysis. CV measurements were carried out at a scan rate of 50 mV/s within a potential window of – 0.2 V to +0.6 V, over two complete cycles.^63^ EIS measurements were conducted at a constant DC potential of 200 mV with a 5 mV AC perturbation, across a frequency range of 1 Hz to 100 kHz.^64^

#### 2.2.4 Surface Modification

Aptamers were used for the selective detection of the CRP biomarker.^65^ To ensure their stability on the electrode surface, they were chemically immobilized to prevent detachment during sample introduction. Each aptamer, comprising a 44-nucleotide sequence, was functionalized with a terminal thiol group. Owing to the strong sulfur–gold affinity, the thiol group readily chemisorbs onto the gold electrode surface, enabling a robust and durable immobilization.^66^

The surface preparation process began with thorough washing of the electrodes using deionized water to remove any contaminants, thereby optimizing the conditions for aptamer stabilization. Subsequently, the aptamers were incubated in a 0.5 mM solution of Tris (2-carboxyethyl) phosphine hydrochloride (TCEP) for 60 min to reduce disulfide bonds and prepare the thiol groups for binding.^67^ Following this reduction step, a 4 μM solution of aptamers was applied to the electrode surface and incubated at a temperature range of 2 to 4 °C for 2 h, ensuring optimal surface coverage. Postaptamer binding, the electrodes were again rinsed with distilled water to remove any nonspecifically bound aptamers. To further enhance surface stability and prevent nonspecific binding, a 2 mM solution of 6-mercapto-1-hexanol (MCH) was used to block vacant sites on the electrode surface. The 4 μL of this blocking solution was added and maintained at 2 to 4 °C for 1 h. A final wash with distilled water was performed to complete the surface modification process, ensuring that the electrode was ready for subsequent analytical applications.

The aptamer employed for C-reactive protein (CRP) detection possessed a specific 44-nucleotide sequence: 5′-/HO-(CH2)­6–S-S-(CH2)­6/GCC TGT AAG GTG GTC GGT GTG GCG AGT GTG TTA GGA GAG ATT GC-3′. It purified using the BIO-RP method, ensuring high purity and optimal performance for biosensing applications.^68^

#### 2.2.5 Preparation of CRP Samples

For the experiments, CRP samples were precisely diluted using phosphate-buffered saline (PBS) to achieve a specific range of concentrations necessary for analysis. The dilutions were prepared to span a concentration gradient from 0.9 to 0.02 ng/mL. To ensure stability and equilibration of the protein in these dilutions, the samples were incubated overnight at a controlled temperature of 4 °C. On the day of the experiment, 4 μL of each prepared CRP solution, at concentrations of 0.9, 0.7, 0.5, 0.3, 0.1, 0.05, and 0.02 ng/mL, were carefully applied to the electrode surface.

#### 2.2.6 Linear Range, Limit of Detection, and Selectivity

The linear range and limit of detection (LOD) are critical parameters in analytical methods, particularly for biosensor performance evaluation. The linear range defines the concentration interval over which the biosensor yields a proportional response to the analyte. In contrast, the LOD refers to the lowest concentration of the analyte that can be reliably distinguished from background noise. Establishing these parameters ensures that the biosensor is appropriate for its intended application, in this case, the detection of C-reactive protein (CRP). The eq 2 was employed to calculate the LOD where SD denotes the standard deviation of the blank (control) signal, and slope (M) is the slope of the calibration curve, representing the sensor’s sensitivity to changes in analyte concentration. The slope (M) was derived from the calibration plot generated using varying concentrations of CRP, and the SD was calculated from blank measurements to represent baseline fluctuations. A lower SD reflects minimal variation and improved detection precision, which is especially important for detecting low concentrations of the target analyte.^69^

$$\mathrm{SD}=\sqrt{\frac{\sum (R_{i}-R_{\mathrm{avg}})2}{n-1}}$$ 1

$$\mathrm{LOD}=\frac{3\times \mathrm{SD}}{M}$$ 2

To evaluate the proposed biosensor’s selectivity against nontarget protein adsorption, such as bovine serum albumin (BSA), interleukin-6 (IL-6), and tumor necrosis factor-alpha (TNF-α), solutions of nonspecific proteins devoid of CRP were used at a dosage of 0.9 ng/mL. A potassium ferro/ferricyanide solution (2 mM) was used as a redox probe, and each experiment was repeated three times.^70^

#### 2.2.7 Performance of the Biosensor with Human Serum

The serum collection and preparation process was conducted with strict adherence to safety protocols. Initially, blood samples were drawn directly from a human’s vein and collected into serum separator tubes (SSTs) that did not contain anticoagulants. These tubes were then left at room temperature for 15 to 30 min to facilitate natural coagulation. During this period, coagulation factors were activated, allowing the blood clotting process to complete fully. After clot formation, the tubes were centrifuged at a speed of 2000 rpm for 5 min using a centrifuge rotor with an approximate radius of 14 cm. This centrifugation effectively separated the blood into three distinct layers. The top layer consisted of serum, a clear, yellowish fluid free from blood cells and coagulation factors. The middle layer is known as the buffy coat. The bottom layer was made up of red blood cells and other cellular components.^71^ The serum was carefully collected from the upper layer of the centrifuged tube, while the remaining contents, including clots and blood cells, were discarded. The collected serum was stored for further analysis. For short-term storage, the serum was kept at temperatures between 2 and 8 °C. For long-term preservation and to prevent any biological or chemical alterations, the samples were frozen at temperatures ranging from −20 to −80 °C.

In this experiment, a technique known as biomarker spiking was employed, where a specific concentration of the biomarker CRP was added to the serum. Initially, human serum free of CRP was diluted 1:100 with phosphate-buffered saline and prepared in various concentrations (0.9, 0.7, 0.5, 0.3, 0.1, 0.01, 0.05, and 0.02) ng/mL. After the addition of CRP, the serum was thoroughly mixed using a gentle vortex to ensure uniform distribution of the biomarker throughout the serum. A blank serum sample (without any spike) was used as a negative control to delineate the differences between it and the spiked samples. Following this preparation, 4 μL of each CRP-spiked serum concentration was applied to the surface of the electrode for electrochemical testing.^72^

## 3 Results and Discussion

The geometric properties of electrodes play a crucial role in capturing electrical signals in biosensors. It is essential to characterize the electrodes to identify differences and determine suitable factors for improvement. This section provides an in-depth analysis of the electrodes and their characterization to facilitate a practical study. Characterization tests were conducted on two distinguished types of electrodes, including TFEs and SPEs. The primary distinctions among the electrodes lie in their size, fabrication method, material composition, and thickness. Here, we focus on the intrinsic characteristics of each electrode type individually before examining their performance collectively in the subsequent section.

### 3.1 Geometrical Differences

FE-SEM imaging was used to examine the geometric and surface characteristics of the electrodes (Figure 2). The fabricated TFEs were generally smaller than the SPEs, with dimensions inspired by recent research. Surface texture differences arise mainly from the fabrication methods.

**Figure 2:**
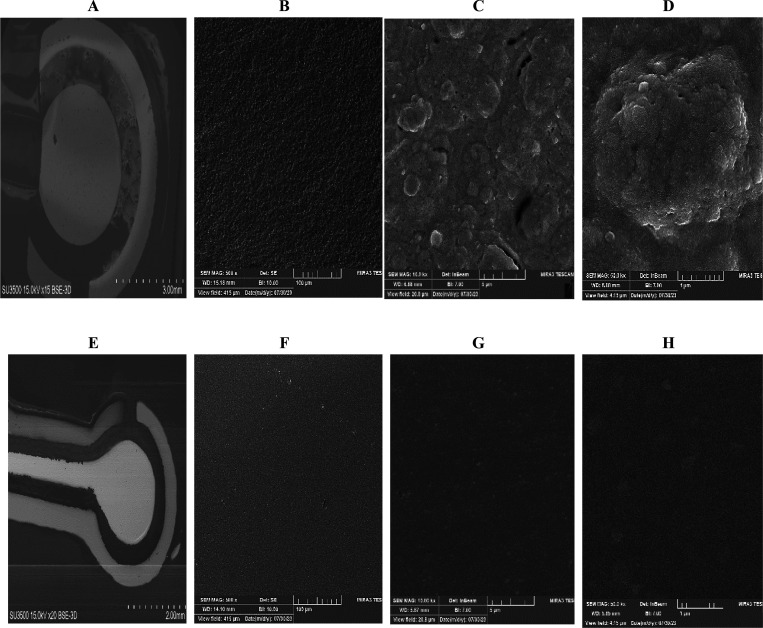
Topographical characterization of the SPE and TFE at different magnifications. (A) Perspective view of the SPE; (B–D) progressively magnified views highlighting the surface roughness of the SPE. (E) Perspective view of the TFE; (F–H) progressively magnified views highlighting the surface roughness of the TFE.

In screen-printed gold electrodes, a paste containing gold nanoparticles is deposited through a 20–70 μm stencil and subsequently baked at high temperature, leaving a layer with a thickness of several micrometers. This process produces a rough surface with pronounced peaks and valleys due to solvent evaporation, while residual nonconductive binders increase the electrode resistivity.^73^

In contrast, TFEs are fabricated via physical vapor deposition (PVD),^74^ using methods such as shadow masking or lithography to deposit a continuous, high-purity metal layer typically less than 1 μm thick. Although the reduced thickness increases sheet resistance relative to SPEs, the uniform and defect-free structure of TFEs enhances intrinsic conductivity and sensitivity.

As shown in Figures 2A–D (SPE) and 2E–H (TFE), SPEs exhibit visibly higher roughness than TFEs. Both electrode types have generally smooth edges without damage from fabrication, though slight irregularities in TFE edges may result from mechanical vibrations during sticker cutting. The thin gold layers in TFEs demonstrate excellent uniformity due to the deposition process,^75^ whereas SPEs have thicker, less uniform gold layers from printed paste. Overall, the smoother and thinner surfaces of TFEs make them more suitable for high-sensitivity electrochemical applications, while the thicker structure of SPEs can provide greater mechanical stability.^76^

AFM resolved nanometer-scale surface roughness over micrometer-scale regions in Figure 3, complementing the FE-SEM observations. Screen-printed electrodes (SPEs) exhibited a pronounced hill-and-valley topography, whereas thin-film electrodes (TFEs) showed a markedly smoother surface with fewer, more dispersed irregularities. Some protrusions observed on the TFE electrodes may originate from environmental particles; however, the measurements were conducted with these features taken into account. The average surface roughness (Sa) was 6.89 ± 4.36 nm (n = 4) for TFEs and 61.15 ± 9.46 nm (n = 4) for SPEs, approximately 9 times higher for SPEs. The test indicates that the average surface roughness (Sa) in SPE is considerably greater than in TFE.

**Figure 3:**
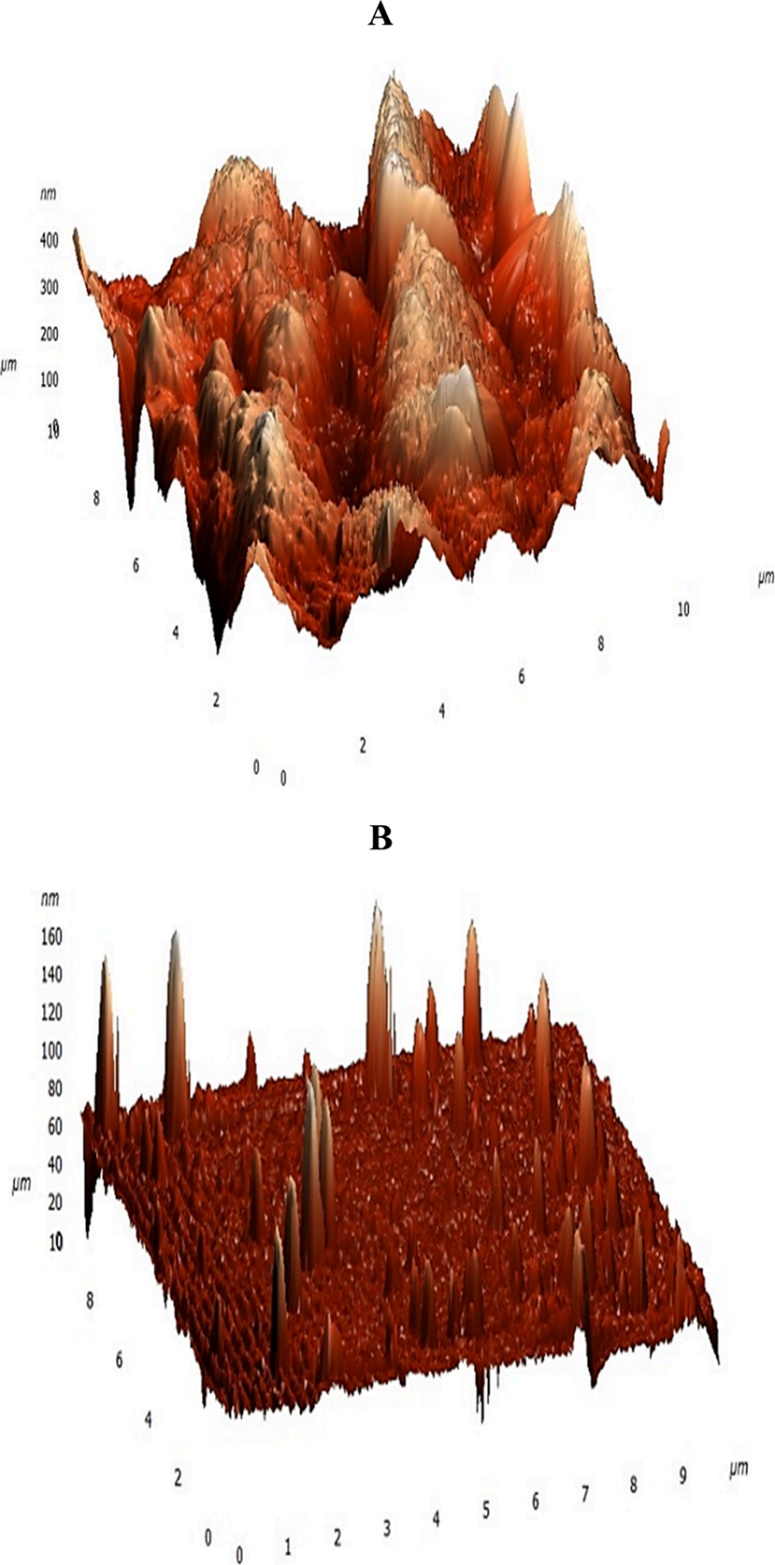
AFM surface topography of both electrodes: (A) SPE and (B) TFE, shown with different height scales for the respective graphs.

Surface characteristics influence charge transfer and the formation of self-assembled monolayers (SAMs). The nonuniform, highly rough SPE surfaces can hinder well-ordered SAM process and introduce local heterogeneity in electron transfer, which may elevate nonspecific adsorption and reduce the differential signal between specific and nonspecific binding. By contrast, the smoother TFE surface provides a more suitable platform for thiol–aptamer immobilization, supporting improved signal-to-noise and better measurement repeatability. Where applicable, surface modification of SPEs can mitigate these effects and enhance sensing performance.^77^

### 3.2 Elemental Composition of Electrodes

The elemental composition of the working electrode (WE) in the SPE system was thoroughly analyzed using EDS data. Elements such as carbon, nitrogen, oxygen, silicon, phosphorus, sulfur, aluminum, and gold were detected in the sample. The main components of the electrode are carbon, oxygen, nitrogen, and aluminum, with atomic percentages of 39.56%, 14.43%, 12.92%, and 12.71%, respectively. The atomic percentages of sulfur, phosphorus, and silicon are 2.76, 6.50, and 6.25, respectively, making them minor components. One of the most critical components in the conductivity of these electrodes is gold, which has the highest weight percentage of 36.14 and an atomic percentage of 4.87. Gold’s high weight percentage indicates its heavy atomic mass compared to other elements. Several components, including silicon, are combined to form a composite in this electrode. The EDS analysis of the working electrode (WE) of the TFE showed that the layer material includes gold and titanium. Titanium has an atomic percentage of 44.12 and a weight percentage of 16.11. Conversely, gold has a weight percentage of 83.89% and an atomic percentage of 55.88%. Titanium serves as an adhesive layer that not only prevents the layers from separating but also strengthens the bond between the gold and the substrate. The high weight percentage of gold (83.89%) indicates that a substantial layer of gold has been deposited on the electrode surface. Figure 4 illustrates the EDS at the surface of the working electrode.

**Figure 4:**
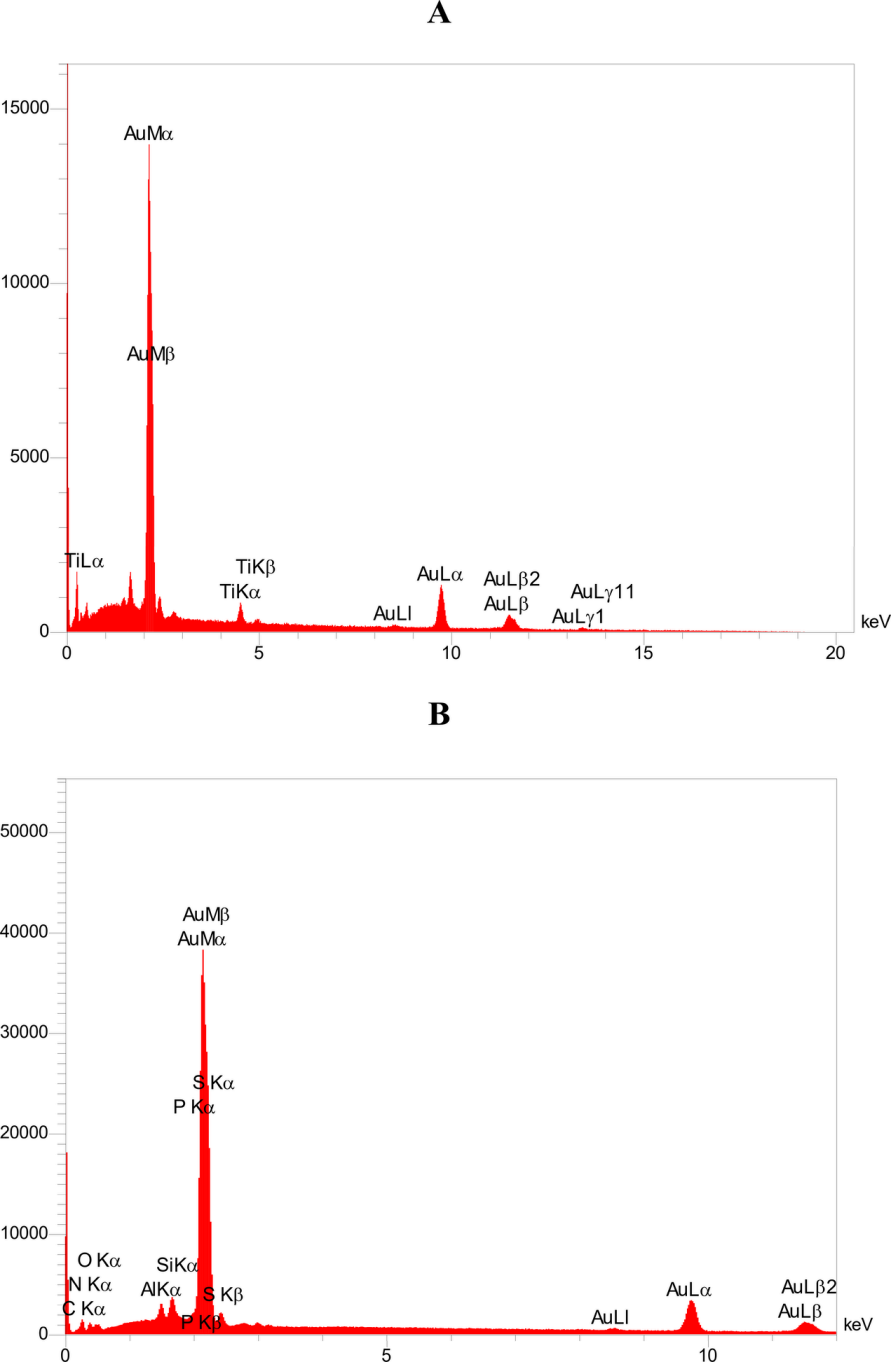
EDS spectra showing elemental composition of the working electrodes: (A) TFE and (B) SPE. The horizontal axis indicates X-ray energy (keV), while the vertical axis represents the intensity of detected X-rays.

In screen-printed electrodes, silver constituted the largest portion of the reference electrode at 77.54 wt %. Other elements present included carbon (C), magnesium (Mg), aluminum (Al), sulfur (S), chlorine (Cl), potassium (K), calcium (Ca), and gold (Au), accounting for 22.46% of the total weight. In contrast, the TFE was composed of only two elements: silver and titanium, which made up 84.04% and 15.96% of the weight, respectively. Figure 5 shows the EDS at the surface of the reference electrode.

**Figure 5:**
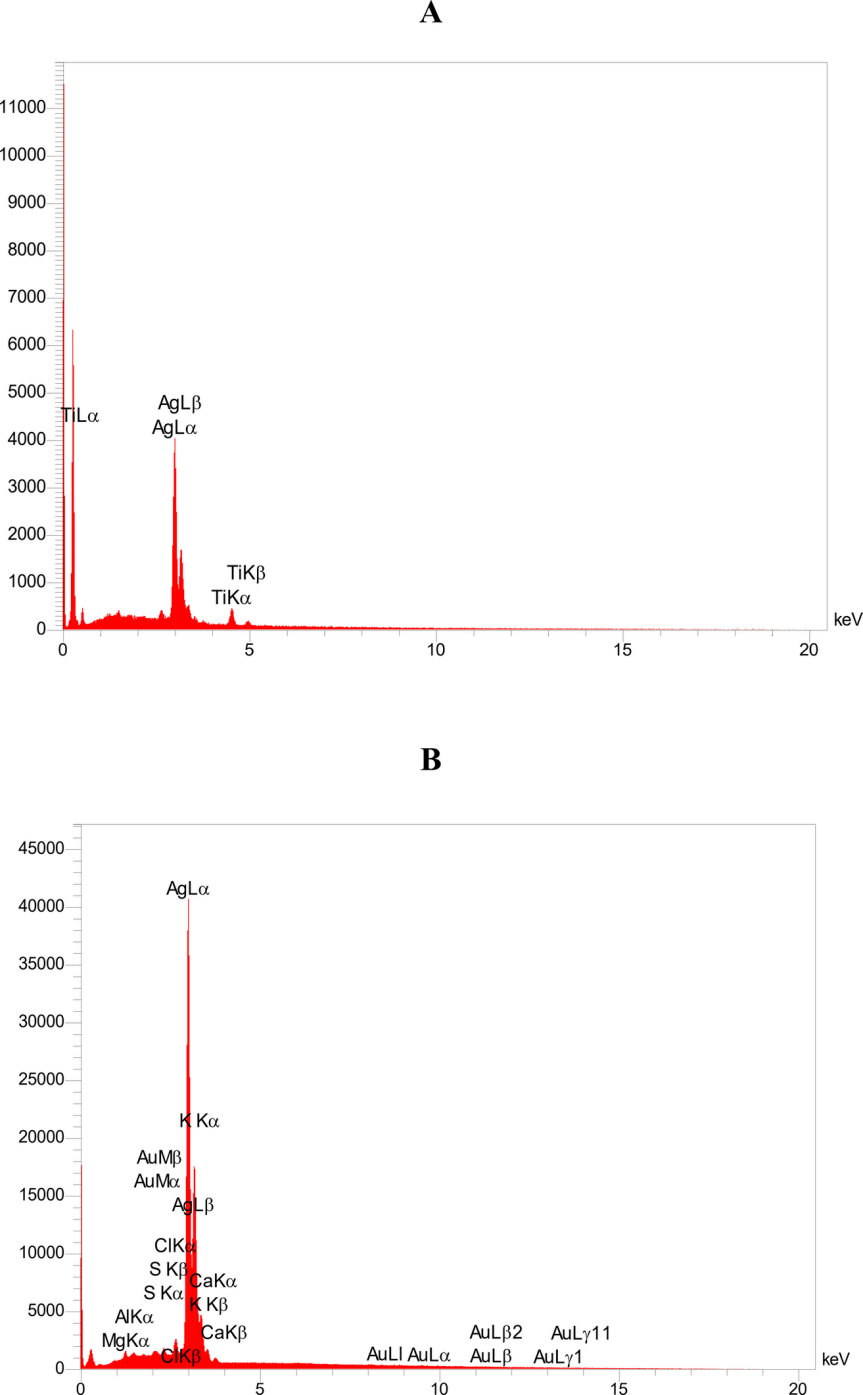
EDS analysis of the reference electrodes, highlighting elemental differences between the (A) TFE and (B) SPE.

Our observation demonstrated that the quantity and type of elements are variable locally, depending on the location of the SPE. Nevertheless, averaging the measurements from three distinct electrode areas could provide a valuable understanding of the relative proportions of various materials. For instance, one investigated location had no sulfur element, while another had 3.48 wt %.

These results indicated that the compositional percentages of the elements in the TFE are uniform across the entire surface, and there are no significant differences between different parts of this electrode. No agglomeration has occurred, and there are no clusters of nonmetals. This surface uniformity, along with its high purity and the absence of different elements on the electrode’s surface, promises the ideal deposition of sensor elements on the designed electrode.

### 3.3 Results of Thickness Analysis

A stylus profilometer determines film thickness by scanning a fine-tipped stylus across the surface and recording its vertical movements under a controlled load. Its primary application is measuring the thickness of various layers. The thickness analysis results presented in Figure 6 determined that the thickness of thin-film gold electrode samples is less than that of commercially printed screen-printed electrodes across all three electrode types and the nanometer range.

**Figure 6:**
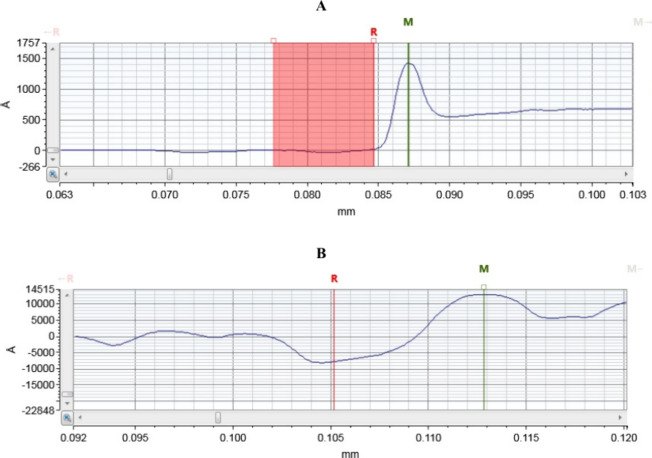
Thickness analysis for (A) TFE electrode and (B) SPE electrode.

The total profile thickness difference calculated is 1400.83 Å (140.08 nm). The *R* position represented the starting point of the gold layer, with a minimal thickness of 29.05 Å (2.90 nm.). The *M* position corresponded to the maximum gold layer thickness, measuring 1429.88 Å (142.98 nm). This reduction in thickness can also present economic advantages and optimizations in specific applications. Additionally, thin-film electrodes offer greater flexibility and ease of manipulation, a characteristic particularly evident in TFEs. In such thin gold films, a smoother surface can reduce electron scattering at surface and grain boundaries, potentially enhancing electron mobility and intrinsic conductivity. The screen-printed electrodes analysis confirmed 20597.03 Å (2059.70 nm thickness) on the substrate, with minimal lateral variation.

At a thickness of 140.08 nm, the film is sufficiently thick to avoid pronounced quantum confinement effects,^78,79^ while remaining thin enough to benefit from its high surface-to-volume ratio.^80^ Furthermore, since the interface is between gold and a titanium substrate, creating a smooth and cohesive interface allows for the control of electrical resistance and electron scattering at the interface boundary.^81^ One potential drawback in thinner samples could be a reduction in structural integrity. However, SEM images showed that in this study, no weakness against damage, deformation, or failure was observed in the TFE samples, as this research was not conducted under harsh environmental conditions or high pressure.

### 3.4 Cyclic Voltammetry (CV) and Electrochemical Impedance Spectroscopy (EIS) Analysis

One of the most significant outcomes of the cyclic voltammetry (CV) tests is the shift in peak current and peak-to-peak potential separation (ΔEp) observed across different electrode modifications. The peak current of the ferro-/ferricyanide redox couple was notably lower at aptamer-functionalized electrodes compared to the bare gold electrode. In the CV curves corresponding to the aptamer-modified surface, both the oxidation and reduction peak potentials shifted more prominently than in the unmodified condition. This increased peak-to-peak potential separation (ΔEp) suggests enhanced complexity in the electron transfer process, likely due to interactions between the immobilized aptamer molecules and the electrode surface.^82^

Quantitatively, the bare gold electrode exhibited a ΔEp of 0.288 V, indicating relatively efficient electron transfer and moderate electrochemical reversibility. After aptamer immobilization (gold/aptamer), ΔEp increased markedly to 0.486 V, reflecting the formation of an electron transfer barrier and the electrostatic repulsion between the immobilized aptamer and the redox couple. Interestingly, the subsequent addition of MCH as a blocking agent to prevent nonspecific binding sites led to a decrease in ΔEp to 0.197 V, indicative of improved interfacial organization and conductivity. Upon exposure to CRP, the ΔEp increased slightly, confirming the biosensor’s ability to detect the presence and rising concentration of the target protein.^83^

The cyclic voltammetry (CV) curves presented in Figure 7 were recorded following the stepwise addition of CRP solutions at concentrations of 0.02 and 0.9 ng/mL in phosphate-buffered saline (PBS) to the biosensor’s working electrode (WE). These results confirm the aptamer’s successful immobilization and the effective blocking of nonspecific sites on the electrode surface to detect varying concentrations of CRP.

**Figure 7:**
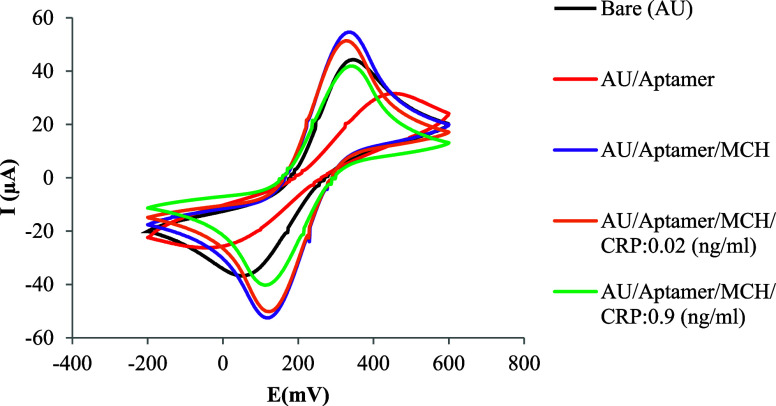
Electrochemical function of the biosensor after surface modification, evaluated using cyclic voltammetry (CV) at a scan rate of 50 mV/s within the potential range of −0.2 to +0.6 V.

The peak-to-peak potential separation (ΔEp) values obtained from cyclic voltammetry measurements demonstrate a consistent trend in response to increasing CRP concentrations in the ferro-/ferricyanide redox couple. Initially, the aptamer/MCH-modified gold electrode (in the absence of CRP) exhibited a ΔEp of 0.197 V. Upon the introduction of 0.02 ng/mL CRP, ΔEp slightly increased to 0.203 V, reflecting the initial formation of aptamer–CRP complexes, which begin to hinder electron mobility. As the CRP concentration increased incrementally to 0.05, 0.1, 0.3, 0.5, and 0.7 ng/mL, the ΔEp progressively rose to 0.210, 0.223, 0.236, 0.241, and 0.249 V, respectively, suggesting increased resistance at the electrode surface due to greater coverage by CRP molecules. At the highest tested concentration (0.9 ng/mL), ΔEp reached 0.257 V, confirming enhanced electrochemical impedance caused by extensive aptamer–CRP binding. This upward shift in ΔEp correlates directly with the increased surface coverage by CRP molecules, leading to higher electrochemical impedance and reduced charge transfer efficiency. This increasing trend is illustrated in Figure 8.

**Figure 8:**
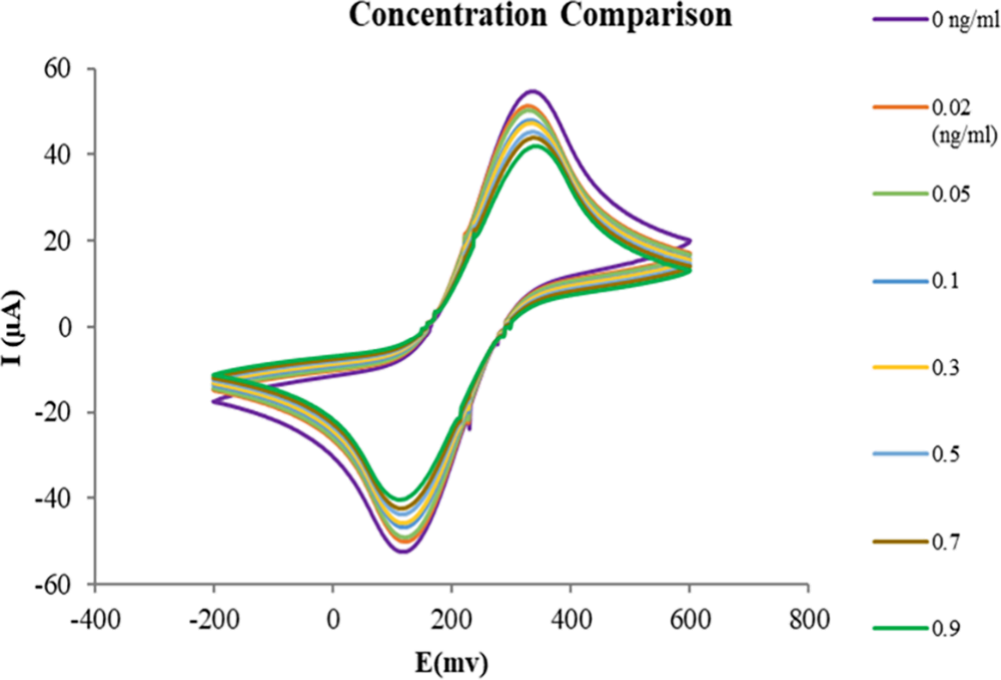
Performance evaluation of the biosensor using cyclic voltammetry (CV) at CRP concentrations of 0.9, 0.7, 0.5, 0.3, 0.1, 0.05, 0.02, and 0 ng/mL. Measurements were conducted at 50 mV/s over −0.2 to +0.6 V.

The Nyquist plot represents impedance data from electrochemical impedance spectroscopy (EIS) measurements, as shown in Figure 9. The horizontal axis corresponds to the real part of the impedance, which is associated with resistance, while the vertical axis represents the imaginary part, which relates to the system’s reactance. The presence of a semicircle in the plot typically indicates a charge transfer process. The diameter of this semicircle is proportional to the charge transfer resistance (Rct); the larger the diameter, the higher the Rct and the slower the electron transfer rate.^84^ In this study, the Nyquist plot illustrates variations in the resistance of the working electrode (WE) during biosensor operation. Upon aptamer immobilization on the WE, Rct arrived at 1.78 kΩ due to surface modifications that reduced the effective sensing area of the electrode. As the CRP concentration increased to 0.02 and 0.9 ng/mL, Rct further increased to 2.0 and 2.8 kΩ, respectively, indicating that CRP binding hindered electron transport.

**Figure 9:**
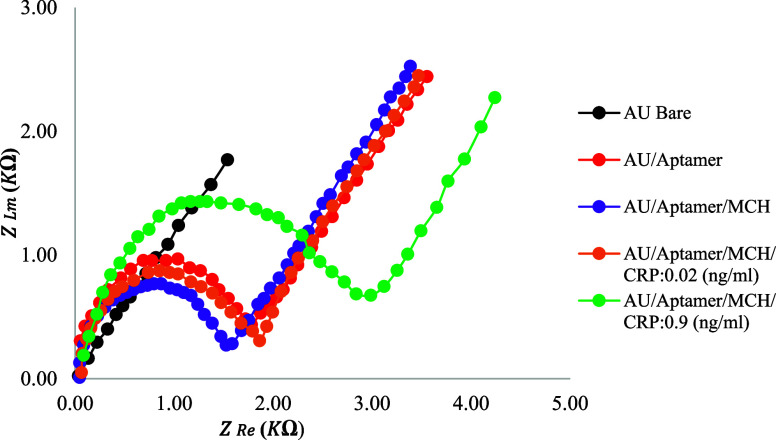
EIS method was applied to measure two different concentrations of CRP.

In addition to evaluating the charge-transfer resistance (Rct), the solution resistance (Rs) was monitored throughout the CRP detection process as an indicator of electrolyte conductivity and the stability of the ohmic path between the working and reference electrodes. Rs is primarily influenced by the ionic conductivity of the electrolyte, the measurement geometry, and electrode contact conditions. In this study, Rs varied within a range: 0.033 kΩ (bare Au), 0.054 kΩ (aptamer), 0.045 kΩ (aptamer/MCH), 0.065 kΩ (0.02 ng/mL CRP), and 0.087 kΩ (0.9 ng/mL CRP), reflecting changes in effective area and contact configuration.

The geometric area of the 2 mm-diameter electrode is 0.0314 cm^2^. Using cyclic voltammetry in 2 mM ferri/ferrocyanide at a 50 mV/s scan rate and the Randles–Ševčík equation, the electroactive area was 0.134 cm^2^ for the clean electrode and increased to 0.165 cm^2^ after thiolated-aptamer modification and MCH blocking. This electroactive area gain is consistent with improved surface accessibility and charge-transfer pathways.The EIS results, reflecting variations in both Rct and Rs, were consistent with the cyclic voltammetry (CV) data.

Calibration curve demonstrating the relationship between CRP concentration and the maximum peak current obtained from cyclic voltammetry (CV) measurements in Figure 10. The CRP concentration (ranging from 0.02 to 0.9 ng/mL) is plotted along the horizontal axis, while the corresponding maximum peak current (in μA) is shown on the vertical axis. A linear regression model was fitted to the data, resulting in the equation y = – 10.043x + 50.701 with a high correlation coefficient (R^2^ = 0.9772), indicating strong linearity. The observed trend confirms that the peak current decreases proportionally with increasing CRP concentration. Based on this calibration curve, the limit of detection (LOD) for CRP was calculated to be 6.5 pg/mL.

**Figure 10:**
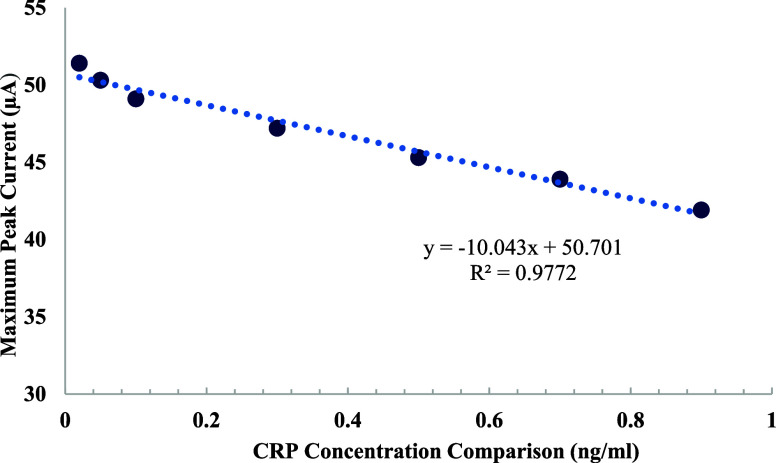
Dose–response calibration curve for C-reactive protein (CRP) detection within the concentration range of 0.02–0.9 ng/mL.

### 3.5 Evaluation of the Biosensor’s Selectivity and Stability

The maximum current (I) was measured for CRP and other nontarget proteins (TNF-α, IL-6, and BSA) at a concentration of 0.9 ng/mL, as shown in Figure 11. In the presence of CRP, a current of 41.9 μA was recorded, indicating specific interactions between CRP and the surface of the sensor. In contrast, in the absence of CRP, the maximum current reached 54.6 μA, representing the baseline response of the sensor without the target protein. TNF-α, IL-6, and BSA produced signals of 52.7 μA, 54.1 μA, and 51.9 μA, respectively. A 2 mM potassium ferro-/ferricyanide solution was used as the redox probe, and all experiments were conducted in triplicate. The peak currents observed for the nonspecific proteins were comparable to those of the CRP-free sample, with variations below 5%, indicating no significant interaction with the sensor. To quantitatively assess the biosensor’s selectivity, the percent current deviation was calculated relative to the CRP signal at 0.9 ng/mL (41.9 μA). The deviations for the CRP-free sample, TNF-α, IL-6, and BSA were 30.3%, 25.8%, 29.1%, and 23.9%, respectively. These significant differences in current confirm that the fabricated biosensor exhibited high specificity for CRP, with minimal interference from nontarget biomolecules.

**Figure 11:**
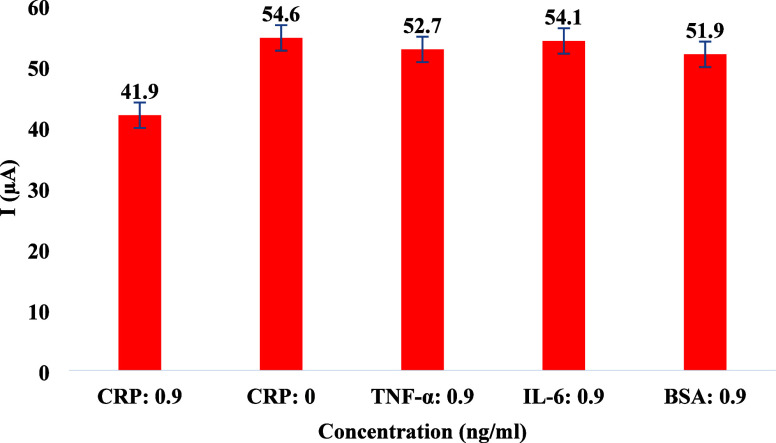
Biosensor’s selectivity was evaluated by comparing its maximum peak current in a CRP-free solution to other protein solutions at a 0.9 ng/mL concentration.

To evaluate the long-term stability of the aptamer-based electrochemical biosensor, signal retention was monitored over a 21-day period. The fabricated electrodes were stored at room temperature and periodically tested for their current response to a fixed CRP concentration (0.9 ng/mL). As shown in Figure 12, the biosensor retained 98.1% of its initial signal after 3 days, 95.7% after 7 days, 94.3% after 14 days, and 92.1% after 21 days. This gradual decline in signal response demonstrates the sensor’s excellent long-term stability and confirms the preservation of both structural and functional integrity of the sensing interface over time.

**Figure 12:**
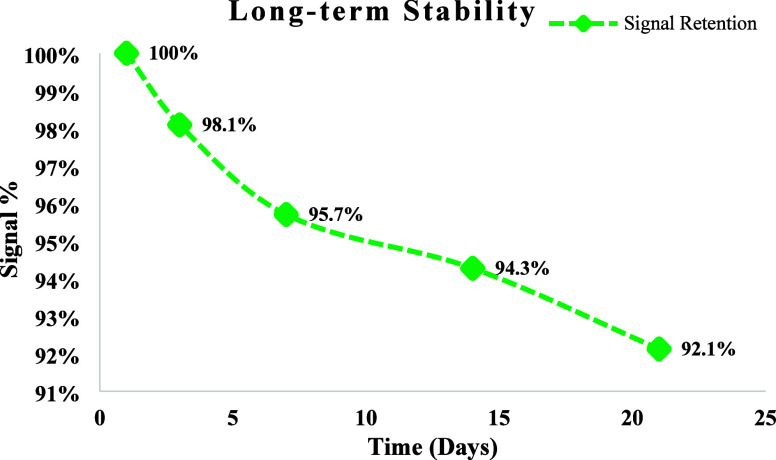
Long-term stability of the fabricated aptamer-based electrochemical biosensor evaluated over 21 days.

### 3.6 Application in the Analysis of Serum Samples

The assembled biosensor was evaluated for its performance in real sample matrices to verify its capability for CRP detection in human serum containing various biomolecules. For this purpose, CRP-free human serum was diluted 1:100 with PBS and used as the sample medium. A series of CRP-spiked samples (4 μL each) with concentrations of 0.02, 0.05, 0.1, 0.3, 0.5, 0.7, and 0.9 ng/mL were applied directly onto the working electrode (WE). Electrochemical measurements were performed using the cyclic voltammetry (CV) technique, and each test was repeated in triplicate. CRP concentrations were quantified by analyzing the maximum peak current responses. As shown in Figure 13, the measured concentrations (0.023, 0.055, 0.12, 0.34, 0.58, 0.78, and 1.12 ng/mL closely matched the spiked values. The relative recovery ranged from 110% to 124%, with relative standard deviations (RSDs) between 0.15% and 3.81%, confirming the biosensor’s reliability and suitability for CRP detection in complex biological samples.^85^

**Figure 13:**
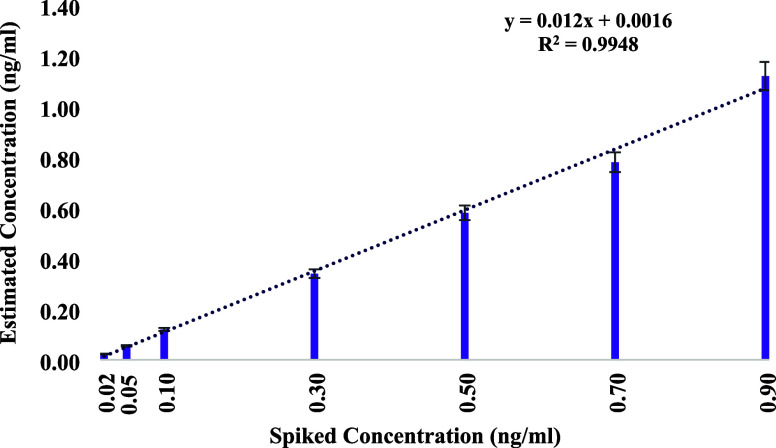
Analysis of real and predicted CRP concentrations using diluted human serum in an aptasensor.

### 3.7 Analytical Evaluation of an Electrochemical CRP Biosensing Platform

Table 1 summarizes the characteristics of electrochemical biosensors previously reported for the determination of CRP. These devices have been fabricated using diverse methodologies and are available in formats such as microwire, disk, thin-film, and screen-printed designs, which may be manufactured from various materials, including gold, carbon, and other conductive substrates. A range of surface receptors, including aptamers, antibodies, and peptides and others, have been employed as biorecognition elements for CRP detection. In the present study, a portable, disposable thin-film gold electrode was fabricated via physical vapor deposition (sputtering) and functionalized with an aptamer immobilized on its surface. The system operated using cyclic voltammetry (CV) and electrochemical impedance spectroscopy (EIS). Aptamers possess several notable advantages, including exceptional high, low cytotoxicity, and ease of synthesis under controlled laboratory conditions. The label-free detection approach further enhances operational simplicity and reduces overall assay costs. Future work will focus on integrating the thin-film electrode with a microfluidic platform to streamline the handling of redox probes, minimize sample and reagent volumes, and improve the device’s suitability for point-of-care applications.

**Table 1:** Summary of Electrochemical Biosensors for CRP Detection: Fabrication Methods, Electrode Designs, and Analytical Performance

fabrication method	electrode type	electrode material	biorecognition element	portable system	measurement technique	linear range (ng/mL)	limit of detection (ng/mL)
microwire assembly^86^	microwire electrode	gold	aptamer-antibody	no	CV, EIS	3125–25 × 10^3^	3125
commercially obtained^87^	standard disk electrode	glassy carbon	aptamer	no	SWV, EIS	0.005–125	0.0017
combined photolithography and electron-beam evaporation^88^	interdigitated wave-shaped microelectrode (thin film)	gold	antibody	yes	EIS	10^–2^–10^4^	0.025
standard thin film fabrication technique^89^	interdigitated electrode (thin film)	gold	antibody	yes	EIS	10^4^–2 × 10^7^	10^5^
commercially obtained^90^	screen-printed electrode	carbon	antibody	yes	CV	2 × 10^3^–10^5^	800
electrodeposition^91^	interdigitated micro comb electrode	gold	antibody	no	EIS	10^–6^–10^–2^	10^–6^
commercially obtained^92^	screen-printed electrode	carbon	aptamer	yes	DPV, SWV	10^–3^–10^2^	2.9 × 10^–4^
commercially obtained^93^	standard disk electrode	gold	peptide	no	CV, SWVEIS	0–36	0.7
commercially obtained^94^	standard disk electrode	gold	antibody	no	CA, EIS	10^2^–2 × 10^4^	3.43 × 10^–4^
physical vapor deposition (this work)	thin film electrode	gold	aptamer	yes	CV, EIS	0.02–0.9	6.5 × 10^–3^

## 4 Conclusions

This work effectively demonstrated a new, simple, and rapid method for fabricating multimaterial thin-film disposable electrodes using a shadow mask technique on both rigid and flexible substrates. In this procedure, a three-electrode system was fabricated on a PMMA substrate. The technique employed readily available PVC sticker film as a disposable shadow mask, eliminating the need for time-consuming and costly photolithography processes. The fabricated thin-film electrodes (TFEs) exhibited excellent characteristics compared to commercially available screen-printed electrodes (SPEs). Characterization using FE-SEM, AFM, and EDS revealed significant differences in surface morphology, roughness surface, and elemental composition between TFEs and SPEs. TFEs demonstrated a remarkably smoother surface, attributed to the physical vapor deposition process, which enables the formation of a thin and uniform conductive layer. The average surface roughness of TFEs was measured at 6.89 ± 4.36 nm, significantly lower than the 61.15 ± 9.46 nm observed for SPEs. This smoother surface enhances electron transfer efficiency.

Additionally, TFEs exhibited higher purity of gold in the working electrode (WE) and silver in the reference electrode (RE) compared to SPEs. The TFEs primarily consisted of titanium and gold for the WE, and titanium and silver for the RE, whereas SPEs contained a mixture of elements, including carbon, oxygen, nitrogen, aluminum, and gold. The presence of nonconductive materials in SPEs increases resistivity and reduces sensitivity. The thickness of the gold layer in TFEs was determined to be 140.08 nm, significantly thinner than the 2059.70 nm thickness observed in SPEs. Electrochemical characterization using cyclic voltammetry (CV) and electrochemical impedance spectroscopy (EIS) confirmed the superior performance of TFEs. The fabricated TFEs were employed in a biosensing experiment for the detection of C-reactive protein (CRP), a critical biomarker for sepsis, to evaluate their performance. In contrast to the minimal response to nontarget proteins, the biosensor exhibited a marked decrease in current upon CRP addition, demonstrating high selectivity toward the target protein. The TFEs demonstrated a linear range of 0.02–0.9 ng/mL and a limit of detection (LOD) of 6.5 pg/mL. Furthermore, tests conducted in human serum samples showed a recovery rate of 110%–124%, with relative standard deviations ranging from 0.15% to 3.81%, confirming the biosensor’s applicability in real-world samples. The results of this study clearly demonstrate the advantages of the developed TFE fabrication method. The combination of excellent electrochemical performance, ease of fabrication, and disposable, TFEs as promising candidates for a wide range of applications, particularly in electrochemical biosensing for the detection of infectious diseases.

## Abbreviations

POC: point-of-care
TFE: thin-film electrode
CRP: C-reactive protein
WE: working electrode
CE: counter electrode
RE: reference electrode
PVC: polyvinyl chloride
PMMA: poly (methyl methacrylate)
Ti: titanium
Au: gold
Ag: silver
FESEM: field emission scanning electron microscopy
AFM: atomic force microscopy
EDS: energy-dispersive X-ray spectroscopy
LOD: limit of detection
μTAS: micro-total analytical systems
IDEs: interdigitated electrodes
FET: field-effect transistors
SML: shadow mask lithography
SPE: screen-printed electrode
PVD: physical vapor deposition
PC: polycarbonate
PEN: polyethylene naphthalate
LOC: lab-on-chip
PCT: procalcitonin
TNF-α: tumor necrosis factor-alpha
IL-6: interleukin-6
PSP: pancreatic stone protein
CV: cyclic voltammetry
EIS: electrochemical impedance spectroscopy
ΔEp: peak-to-peak potential separation
Rct: charge transfer resistance
Rs: solution resistance
